# Porous Film Coating Enabled by Polyvinyl Pyrrolidone (PVP) for Enhanced Air Permeability of Fabrics: The Effect of PVP Molecule Weight and Dosage

**DOI:** 10.3390/polym12122961

**Published:** 2020-12-11

**Authors:** Jiantang Jiang, Yifeng Shen, Deyou Yu, Tao Yang, Minghua Wu, Lei Yang, Michal Petru

**Affiliations:** 1 Engineering Research Center for Eco-Dyeing and Finishing of Textiles, Zhejiang Sci-Tech University, Hangzhou 310018, China; jiantangjiang@163.com (J.J.); shenyf66@sina.com (Y.S.); yanglei@zstu.edu.cn (L.Y.); 2 Key Laboratory of Advanced Textile Materials and Manufacturing Technology, Ministry of Education, College of Materials and Textiles, Zhejiang Sci-Tech University, Hangzhou 310018, China; 3 Institute for Nanomaterials, Advanced Technologies and Innovation, Technical University of Liberec, 461 17 Liberec, Czech Republic; tao.yang@tul.cz (T.Y.); michal.petru@tul.cz (M.P.)

**Keywords:** porous film, morphology, etching, polyvinyl pyrrolidone, air permeability

## Abstract

This study developed a versatile and facile method for creating pores and tuning the porous structure in the polymer latex films by selectively etching the added functional polyvinyl pyrrolidone (PVP) molecules. The pore formed in the latex films had a similar morphology to that of PVP aggregation before etching. This observation promotes us to regulate the pore morphology that determines the film’s property, such as air permeability through varying the PVP molecule weight and dosage. To this end, the effects of PVP molecule weight and dosage on the pore formation were systematically studied. The results showed that the average pore size of porous film decreased from >10 μm to sub-micron (about 0.4 μm) as the molecular weight or the dosage of PVP increased. This was ascribed to the strong adsorption affinity of PVP molecule onto the latex particle surface, which further hindered the diffusion and self-assembly of PVP molecule. In addition, this interaction became much stronger when the higher molecule weight of PVP or the higher dosage of PVP was employed, leading to the decreased size of PVP aggregation, as well as the formed pores in the latex films. Furthermore, the addition of PVP had little effect on the color of coated fabric based on the results of CIE L*a*b* measurement. The proposed facile method can be used to improve the air permeability of coated fabrics.

## 1. Introduction

Polymer latex derived from emulsion polymerization is widely used in many industries such as binders, textile finishing, paper coating, architectural and pharmaceutical industries, because it can largely reduce the volatile organic compound (VOC) emission [1,2,3,4,5,6,7,8,9]. In particular, technical/smart textiles can be facilely developed by casting functional polymer latex into conventional textiles [10,11,12,13,14,15,16,17,18,19,20]. In this procedure, film formation is an important procedure for the use of commercial latex. Typically, the latex is allowed to be casted into substrates, such as textiles and glasses, to form a desirable film.

In some cases, the film is easily designed as a non-porous “wall” barrier so that undesirable impurities can be restricted [21,22,23,24]. In contrast, other applications in separation, drug or nutrient release, and catalysis need porous films to achieve controllable permeation, which remains a big challenge in facilely regulating the pore structures [25,26,27,28,29,30,31]. To this end, several strategies have been devised to prepare the porous film by casting the aqueous latex into certain substrates.

Adopting the drying temperature close to the minimum film formation temperature (T_mff_) during film curing is one of the most used strategies. In this case, the latex particle suffers an incomplete deformation and thus promotes the formation of pores in the polymer film [32,33]. Another widely used strategy is to introduce a pore forming agent into the latex. The selected agent can be incorporated into the latex film during film formation and then is removed by selectively etching. Examples of pore forming agents tested include small molecule additives (e.g., urea, glycerol, glucose, sucrose, etc.) [34,35,36,37,38,39] and water-soluble polymers (WSPs) (e.g., polyvinyl pyrrolidone (PVP), polyethylene glycol (PEG), etc.) [40,41,42]. Since small molecule additives have limitations in controlling over the pore structure of film, WSPs having the potential to tune the pore morphology due to their greater incompatibility with the substrate are an ideal candidate for porous film production. The preparation of the porous film usually involves organic solvents in the casting systems. The organic solvent enables the well dissolution of used polymers to form a homogeneous casting solution. The coating film on the certain substrate using this casting solution can form pores after a period of solvent evaporation or solvent dissolution [43,44,45]. However, the organic solvent probably discharges VOC and other harmful substance, posing negative effect on the ecological environment and human-being health. Therefore, it is necessary to explore water-based porous film formation methods to avoid the use of organic solvents.

Our earlier works have demonstrated the possibility of using PVP as pore forming agent for preparing the water-based porous latex film [46,47]. However, the regulation of the pore morphology in the latex film forming system remains a challenge. In this study, the pore morphology of latex films, using PVP as pore forming agent, was systematically investigated to gain a deep insight into the regulation principle. The main objectives of our study are; (i) exploring the effect of PVP molecule weight and dosage on the pore morphology evolution; and (ii) applying the optimized process of proposed method to enhance the air permeability of coated fabrics. Our study can pave the way toward the understanding of pore formation using WSPs as an additive and provide a facile coating method for enhancing the air permeability of fabrics.

## 2. Experimental

### 2.1. Materials

Styrene-butyl acrylate copolymer latex with a solid content of about 30% was purchased from Zhejiang Kefeng Silicone Co., Ltd., Jiaxing, China. The received latex has an average particle size of 90 nm and a distribution index of 0.05. Polyvinyl pyrrolidone K30 (PVPK30, number average molecular weight of 10,000 g mol^−1^) and Polyvinyl pyrrolidone K15 (PVPK15, number average molecular weight of 2500 g mol^−1^) were purchased from Hangzhou Nanhang Industrial Co., Ltd., Hangzhou, China. Synthetic thickener KF-386 was purchased from Zhejiang Kefeng Silicone Co., Ltd., Jiaxing, China. Methanol (CH_3_OH) was obtained from Shanghai Macklin Biochemical Co., Ltd, Shanghai, China. The water utilized in all experiments was deionized water. The used fabric was a plain woven and 100%-dyed cotton fabric. The mass density and yarn size of the used cotton fabric were 125 ± 2 g m^−2^ and 18.2 tex, respectively. The density of fabric in the warp and weft direction were 10.8 and 5.4 threads cm^−1^, respectively. The fabric was obtained from Zhejiang Haoyu Technology Co., Ltd, Shaoxing, China.

### 2.2. Preparation of Latex Film

The emulsion was prepared by mixing the styrene-butyl acrylate copolymer latex and PVP, whose dosage was calculated based on the dry mass of the styrene-butyl acrylate copolymer latex. First, a certain amount of PVP was dissolved in water to form an aqueous solution A. Then, the solution A was added into the weighed latex to prepare the emulsion under stirring at 250 rpm for 10 min. The detailed formula of emulsion was summarized in Table 1.

The prepared emulsion was poured into the PTFE vessel and dried in an oven at a temperature of 50 °C. Then the formed film was removed from the PTFE vessel and was re-equilibrated to the ambient conditions for 10 min. Afterwards, the film was immersed into a methanol/water (weight ratio = 10:90) solution and kept for 10 min to promote the pore formation by dissolving PVP. In the end, the porous film was filtered and dried at room temperature [46,47].

### 2.3. Coating of Cotton Fabric

The preparation of the coating paste was described as follows: A mixture of thickener KF-386 (0.5 g) and the water (49 g) were stirred at 1600 rpm for 30 min. At the same time, PVPK30 dissolved in water and mixed well with BA. Then the two mixtures were mixed and stirred at 1200 rpm for 30 min. The formula of the coating paste was summarized in Table 2. The cotton fabric was coated with the different paste by a knife over roll coating method using a laboratory coating machine (LTE-S, Mathis, Switzerland). The coated cotton sample was soaked into the methanol/water (weight ratio = 10:90) solution for 5 min at room temperature, and then followed by water washing and dried at 160 °C for 3 min.

### 2.4. Characterization

#### 2.4.1. Gravimetric Analysis of Water Evaporation from Latex

The weight loss of the latex dispersion was quantified by a gravimetrical method. The PTFE vessels withdraw from the oven were weighted at intervals of 10 min and putted back immediately. The latex drying kinetic was calculated according to our early study [47]. To clarify the change of drying rate during the film forming process of latex, two parameters were defined. X represented the evaporation of water per unit area at a certain moment, which was calculated from Equation (1), Y represented the latex volume fraction or solid content of latex at a certain moment, which was calculated from Equation (2). First, X was used as the ordinate and dry time was used as the abscissa to draw the graph. Then, the derivative was obtained, and the absolute value of the derivative obtained means the weight of water loss per unit area per unit time. Taking this as the ordinate, and use Y as the abscissa to draw a graph to obtain the curve of the rate of water evaporation with the latex volume fraction,

(1) $$X=\frac{m_{1}-m_{t}}{\pi \;\emptyset^{2}}$$

(2) $$Y=\frac{(m_{n}-m_{0})/1.05}{m_{t}-m_{0}-0.05(m_{n}-m_{0})/1.05}$$

where, ∅ means the radius of the circular concave surface in the PTFE vessel (m); $m_{0}$ means the weight of the PTFE vessel (g); $m_{1}$ means the initial weight of the PTFE vessel after adding emulsion (g); $m_{t}$ means the weight of the PTFE vessel and emulsion after drying for a certain period (g); $m_{n}$ means the weight of the PTFE vessel and polymer after completely dry (g).When the amount of PVP addition was 0%, 5%, 10%, 15%, 20%, 25%, and 30%, 1.05 and 0.05 in Equation (2) were replaced with 1 and 0, 1.05 and 0.05, 1.1 and 0.1, 1.15 and 0.15, 1.2 and 0.2, 1.25 and 0.25, and 1.3, and 0.3, respectively.

#### 2.4.2. Latex Film Morphologies

The dried latex films before washing were prepared with an ultra-thin microtome under the condition of liquid nitrogen freezing and were collected onto the 200 mesh copper wire mesh. Then, the morphology of ultrathin section of the latex films was observed by a transmission electron microscope (TEM, JEM 1230, JEOL, Chiyoda, Japan). The dried latex films before and after washing were quenched in liquid nitrogen, and the surfaces were gold-plated. Then, the cross sections of the films before and after washing were obtained by a scanning electron microscope (SEM, JSM-5610LV, JEOL, Chiyoda, Japan). The pore sizes in the porous latex films were obtained by manually counting 300–400 dispersed phases by microscopic imaging software MiVnt (Shanghai Hui Tong Optical Instrument Co., Ltd., Shanghai, China).

#### 2.4.3. The Performance of Coated Fabric

The air permeability of the coated fabric was measured according to GB/T5453. The CIE L*a*b* of coated fabric was assessed by a color matching instrument (Datacolor SF 600, Lawrenceville, NJ, USA). The crocking fastness and laundering fastness of printed fabrics were determined according to AATCC 8 and AATCC 61 (Test No. 2A) standards, respectively.

## 3. Results and Discussion

### 3.1. Morphologies of Latex Films with PVP

**Figure 1:**
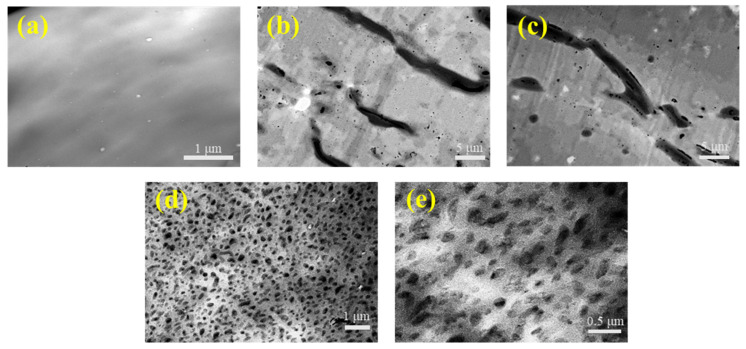
TEM image of ultrathin cross sections of films: (**a**) without using PVP; (**b**) using 5% of PVPK30; (**c**) using 10% of PVPK30; (**d**) using 15% of PVPK30; and (**e**) using 20% of PVPK30.

The morphology of ultrathin section of latex films is displayed on Figure 1. The transmission electron microscopy images (TEM) images in Figure 1 indicated that the pore morphologies in latex films varied significantly as PVPK30 dosage changed. As shown in Figure 1a, the latex film without PVP had a homogeneous structure without pores. After the addition of PVPK30, two distinct phases were observed in the films, where PVPK30 occurred in the dispersed phase and appeared as dark areas in the TEM images (Figure 1b–e). When the PVPK30 dosages were 5% (Figure 1b) and 10% (Figure 1c), strip-like PVPK30 phases with an average length of 10 μm were obviously detected. As the dosage of PVPK30 increased to 15% (Figure 1d), a remarkable decrease in the phase size (an average length of 0.40 μm) was observed. In Figure 1e, a further increase in the dosage of PVP to 20%, however, only caused a very slight decrease in the dispersed phase size to about 0.35 μm.

**Figure 2:**
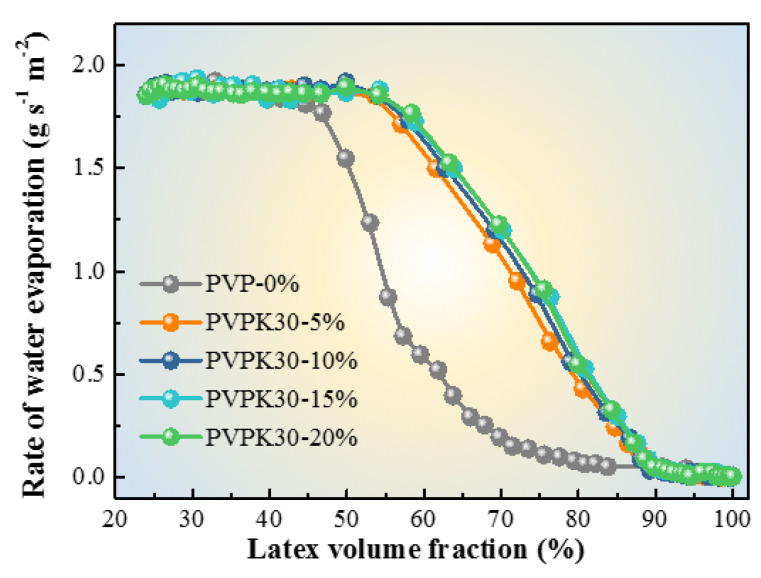
The influence curve of PVPK30 dosage on the drying rate of latex.

To explore the reasons for the dependence of film morphologies on PVPK30 dosages, the film formation processes of latex containing PVPK30 were studied by measurement of latex drying rates. As revealed by the drying kinetic curves in Figure 2, all the film formation processes obeyed a “Three-Stage” model [48,49,50,51,52,53,54]. Taking the case of latex without adding PVP as an example, the first stage held about a constant water evaporation rate of 1.9 g s^−1^ m^−2^ and ended at about 44% latex volume fraction. The second stage proceeded with a gradually deceased evaporation rate till 85% latex volume fraction, which was followed by the onset of the third stage with an extremely low water evaporation rate. In comparison, for the latex with PVPK30, two different points were discerned in the drying kinetic curves.

First, the ending of the first stage delayed to a latex volume dosage as high as 55%, which was independent on the PVPK30 dosage. This observation indicated that PVPK30 improved the colloidal stability, thus ensuring that the latex could keep a constant water evaporation area till 55% latex volume fraction [55,56]. Kellaway and Najib et al. [57] reported that the added PVPK30 molecules were prone to adsorb onto the surface of the latex particles for stabilization. Further research revealed that the saturation point of adsorption was 2.2 × 10^−17^ g nm^−2^. To this end, it was inferred that PVPK30 added into the latex almost adsorbed onto the latex particle surface and provided the latex particles with steric barriers, thus additionally stabilizing the latex in the coating suspension. Second, the drying rate increased as the dosage of PVPK30 increasing during the intermediate stage, demonstrating that the latex with PVPK30 addition had increased areas for water evaporation. This observation could be ascribed to following reasons: After the occurrence of irreversible contact between latex particles, the PVPK30 molecules would desorb from the particle surface and dissolve into the water phase. As a result, the viscosity of water phase would increase and become higher with the increase of PVPK30 dosage. If the viscosity was high enough, the packing of latex particles would be suppressed, leading to loose packing of particles. Therefore, on the one hand, PVPK30 microdomains with submicron size in the final films could be produced and observed (Figure 1d,e). On the other hand, the high viscosity could ensure the latex with a high drying rate owing to the increased “water-air” interfaces for water evaporation. In addition, when the viscosity of water phase was in the low range (e.g., 5% or 10% PVPK30 were applied.), the particle packing process would be little suppressed. The particles could accumulate well and pack densely. In this case, PVPK30 molecules in the aqueous phase could form “strip-like” morphologies with large size (Figure 1b,c) through self-assembly. These observations implied the possibility of regulating the morphologies of PVPK30 by changing the viscosity of the aqueous phase. Except the dosage of PVP as mentioned above, the viscosity is also dependent on the molecular weight of PVP. Therefore, another series of experiments were designed to examine this possibility, where PVPK30 was substituted by PVPK15.

**Figure 3:**
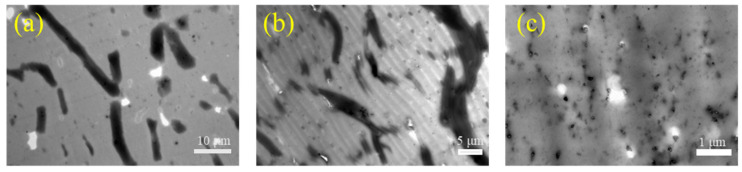
TEM image of ultrathin cross sections of PVPK15: (**a**) using 15% of PVPK15; (**b**) using 25% of PVPK15; and (**c**) using 30% of PVPK15.

**Figure 4:**
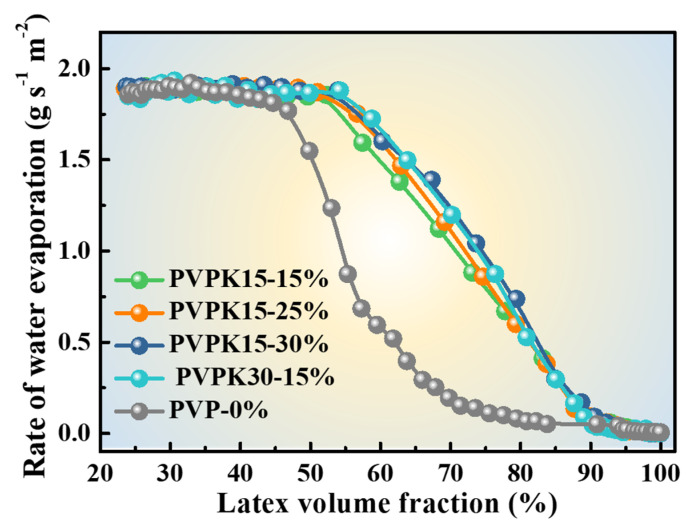
The influence curve of PVPK15 and PVPK30 dosage on the drying rate of latex.

PVPK15 had a lowered molecular weight than PVPK30. It was expected that by decreasing the average molecular weight, the viscosity of the aqueous phase would decrease, thereby enlarging the size of PVP phase and final pore size. The TEM images of films prepared using PVPK15 are shown in Figure 3. When PVPK15 dosage was 15%, the PVPK15 domains in Figure 3a appeared as strip-like shape with an average length of 15 μm, while PVPK30 domains were only 0.40 μm (Figure 1d). The further increase of PVPK15 dosage to 25% (Figure 3b) did not induce any obvious variation of the morphologies except for a decrease in the size. The size of PVPK15 phase decreased significantly as the PVPK15 dosage increased to 30%. As shown in Figure 3c, the average size decreased to around 0.30 μm. By comparison, the average size of PVPK30 phases decreased to about 0.35 μm at an even lower dosage of 20%. The difference in PVP morphologies was coincided with the difference in drying kinetics between latex with PVPK15 and latex with PVPK30. As shown in Figure 4, the latex contained the same dosage of PVPK15 and PVPK30, but the latex with 15% of PVPK15 had a lower drying rate than that with 15% of PVPK30 during the intermediate stage. As discussed above, the lower rate was due to the smaller “water-air” interface area for evaporation because of the larger domain size of PVPK15. When the domain size of PVPK15 decreased, the drying rate of the corresponding latex increased accordingly. As shown in Figure 1e and Figure 3c, the PVPK15 domains decreased to about the same size as that of PVPK30, being just expected, the drying rates of the two latex were nearly the same.

### 3.2. Pore Architecture of Latex Films after Removal of PVP

**Figure 5:**
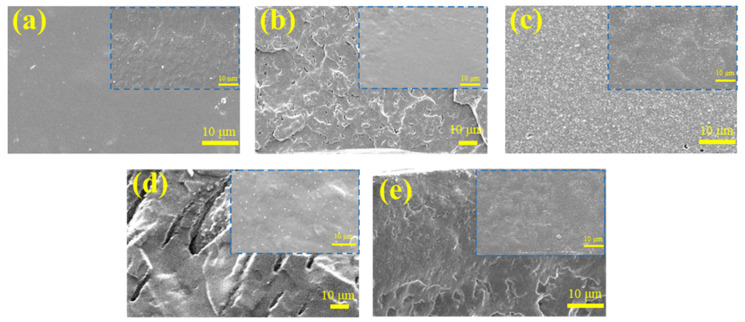
The SEM images of the cross-section of latex film with PVP before (inset images) and after washing: (**a**) using 0% of PVP; (**b**) using 10% of PVPK30; (**c**) using 15% of PVPK30; (**d**) using 15% of PVPK15; and (**e**) using 30% of PVPK15.

The latex films were washed with the mixture of methanol and water with the purpose to remove PVP and realize the porous structure. The SEM analysis was used to confirm the feasibility of the method. The obtained SEM images of washed films are shown in Figure 5. The insets in Figure 5 showed that all the latex films had continuous structure, which was similar to the morphology before washing. The effects of PVP on the film structure were unobvious before washing. After washing, great differences in morphologies were discerned between latex films with and without PVP. As shown in Figure 5a, the film prepared without PVP still resembled the morphology without pores after washing. In contrast, solvent-washing gave rise to a great number of pores in the films prepared with PVP.

The pore sizes decreased from 10 μm to 9.5 μm and futher to 0.4 μm as the PVPK30 dosage increased from 5% to 10%, and to 15%. The introduction of PVPK15 had a very similar effect on the pore sizes. As shown in Figure 3, the pore size decreased from 13.0 μm to 0.4 μm with the growing PVPK15 dosage from 15% to 30%. Either the changing pattern or the pore size agreed well with those of the PVP phases in the films. It was demonstrated that the pores were formed as a result of the removal of PVP.

### 3.3. The Performance of Coated Fabrics

**Figure 6:**
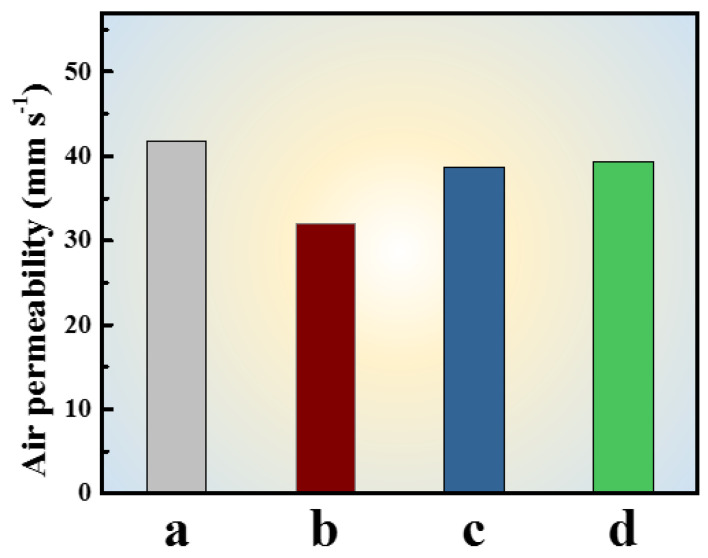
The air permeability of fabrics with different paste: (**a**) original fabric; (**b**) coated fabric without adding PVP; (**c**) coated fabric with adding 10% of PVPK30; (**d**) coated fabric with adding 20% of PVPK30.

After being applied onto the textiles, the polyacrylate latex often forms dense films to ensure the textile dyeing and finishing auxiliaries adhere firmly to the fabric surface. However, it usually leads to a decreased air permeability, and affects the thermal insulation and comfort of the coated fabrics. The porous films may be a good way to improve the air permeability of the final fabrics. The effect of porous films prepared with (or without) PVP on the air permeability of fabric is shown in Figure 6.

It could be seen that the air permeability of the original fabric was about 41.8 mm s^−1^, whereas the permeability of coated fabric without added PVP decreased significantly to about 30 mm s^−1^. By contrast, the permeability of coated fabric with adding PVP gained remarkable improvement. The air permeability of coated fabric could reach to ~39 mm s^−1^, which accounted for ~90% recovery of air permeability. Furthermore, the air permeability of coated fabric with adding 10% of PVPK30 was almost the same as that adding 20% of PVPK30, although the pore sizes produced by 10% of PVPK30 were larger than those by 10% of PVPK30. In other words, the dosage of PVPK30 had little influence on the air permeability of the coated fabrics. This might be because that the pore size became smaller as the dosage of PVPK30 increased, but the number of pores increased, and then the total surface area of the pores did not suffer a big change. In addition, most textiles were colored and required certain color fastness. The effect of coating process on the property of colored fabrics using different paste is shown in Table 3 and Table 4. In Table 3, after adding polyacrylate latex, the color depth of the fabric decreased slightly, but the color difference suffered little changes. When adding PVP as an additive, the color depth of the fabric continued to decrease, and the shade changed slightly, but the color difference from the original fabric was less than 1.00, which was acceptable in the color difference. Further, PVP dosage had little effect on color change. It could be seen from Table 4 that the coated process with or without adding PVP had little effect on the color fastness of coated fabric.

## 4. Conclusions

In summary, this study demonstrated the facile preparation of the porous films using aqueous latex with addition of PVP. The pore morphology in the latex films could be regulated by varying the dosage or the molecular weight of PVP. After the addition of PVP, the film formation processes of all latex still obeyed the “three-stage” model, while the PVP had different influences on the first and second stages. The first stage was not completed until the latex volume fraction reached to a level as high as 55%, which was independent on the PVP dosage. In the second stage, increasing the dosage of PVP would accelerate the water evaporation rate, resulting in the formation of smaller pores. By comparison to original coated fabric (0.2 g s^−1^ m^−2^), the water evaporation rate could reach up to as high as 1.2 g s^−1^ m^−2^ at the latex volume fraction of 70% when PVPK30 was applied. In addition, the added PVP could adsorb onto the latex particle surface and thus improve the latex particle stability. The high viscosity of the aqueous phase formed by the addition of PVP might be responsible for the decrease in the pore size in the film. To this end, the permeability of coated fabric with the addition of PVP in the latex was significantly improved from 32 to 39.4 mm s^−1^. In addition, the color change of coated fabrics was little affected by the addition of PVP. Our study can pave the way toward the understanding of pore formation using WSPs as an additive and provide a facile coating method for enhancing the air permeability of fabrics.

## Figures and Tables

**Table 1:** The detailed formula of emulsion.

Samples	Styrene-butyl Acrylate Copolymer Latex (g)	PVP (g)	Water (g)
PVP 0%	4	0.00	0.8
PVP 5%	4	0.06	0.8
PVP 10%	4	0.12	0.8
PVP 15%	4	0.18	0.8
PVP 20%	4	0.24	0.8
PVP 25%	4	0.30	0.8
PVP 30%	4	0.36	0.8

**Table 2:** The formula of coating paste.

Samples	Thickener KF-386 (g)	Styrene-butyl Acrylate Copolymer Latex (g)	PVPK30 (g)	Water (g)
coated fabric with PVP 0%	0.5	4	0.00	95.50
coated fabric with PVPK30 10%	0.5	4	0.12	95.38
coated fabric with PVPK30 20%	0.5	4	0.24	95.26

**Table 3:** Comparison of the color change with different paste.

Fabric	CIE L*, a*, b*			DE*
	L*	a*	b*	
original fabric	35.11	57.47	8.21	
coated fabric with PVP 0%	35.31	57.21	8.07	0.35
coated fabric with PVPK30 10%	35.71	57.79	7.93	0.74
coated fabric with PVPK30 20%	35.76	57.67	7.77	0.82

**Table 4:** Comparison of the fastness of coated fabric with different paste.

Fabric	Crocking Fastness/Grade		Laundering Fastness/Grade Color Change
	Dry	Wet	
original fabric	4.5	4	4
coated fabric with PVP 0%	4.5	4	4
coated fabric with PVPK30 10%	4.5	4	4
coated fabric with PVPK30 20%	4.5	4	4

## References

[1] Chen Z.J., Hu X.D., Wang X.H., Xiang Z. (2020). The morphology of poly(terminal vinyl dimethicone-co-methyl methacrylate-co-n-butyl acrylate)/pigment composite film and its application in pigment printing of polyester fabric. RSC Adv..

[2] Gao D., Liang Z., Lyu B., Feng J., Ma J., Wei Q. (2016). “Soft” polymer latexes stabilized by a mixture of zinc oxide nanoparticles and polymerizable surfactants: Binders for pigment printing. Prog. Org. Coat..

[3] Gonzalez-Alvarez M.J., Paternoga J., Breul K., Cho H.J., Roshandel M.Z., Soleimani M., Winnik M.A. (2017). Understanding particle formation in surfactant-free waterborne coatings prepared by emulsification of pre-formed polymers. Polym. Chem..

[4] Gonzalez-Martinez J.F., Falk Y.Z., Bjorklund S., Erkselius S., Rehnberg N., Sotres J. (2018). Humidity-Induced Phase Transitions of Surfactants Embedded in Latex Coatings Can Drastically Alter Their Water Barrier and Mechanical Properties. Polymers.

[5] Gurnani P., Perrier S. (2020). Controlled radical polymerization in dispersed systems for biological applications. Prog. Polym. Sci..

[6] Kwon S., Oh K., Shin S.J., Lee H.L. (2020). Effects of hydroxyethyl methacrylate comonomer in styrene/acrylate latex on coating structure and printability. Prog. Org. Coat..

[7] Li L., Wang R., Lu Q. (2018). Influence of polymer latex on the setting time, mechanical properties and durability of calcium sulfoaluminate cement mortar. Constr. Build. Mater..

[8] Nakanishi E.Y., Cabral M.R., Gonçalves P.d.S., Santos V.d., Savastano Junior H. (2018). Formaldehyde-free particleboards using natural latex as the polymeric binder. J. Clean. Prod..

[9] Rodrigues L.D.A., Hurtado C.R., Macedo E.F., Tada D.B., Guerrini L.M., Oliveira M.P. (2020). Colloidal properties and cytotoxicity of enzymatically hydrolyzed cationic starch-graft-poly(butyl acrylate-co-methyl methacrylate) latex by surfactant-free emulsion polymerization for paper coating application. Prog. Org. Coat..

[10] Abdelrahman M., Khattab T. (2019). Development of One-Step Water-Repellent and Flame-Retardant Finishes for Cotton. ChemistrySelect.

[11] Abdelrahman M.S., Fouda M.M.G., Ajarem J.S., Maodaa S.N., Allam A.A., Khattab T.A. (2020). Development of colorimetric cotton swab using molecular switching hydrazone probe in calcium alginate. J. Mol. Struct..

[12] Camlibel N.O., Avinc O., Arik B., Yavas A., Yakin I. (2019). The effects of huntite-hydromagnesite inclusion in acrylate-based polymer paste coating process on some textile functional performance properties of cotton fabric. Cellulose.

[13] Ibrahim N.A., Eid B.M., Khalil H.M., Almetwally A.A. (2018). A new approach for durable multifunctional coating of PET fabric. Appl. Surf. Sci..

[14] Markus M., Speck T., Speck O., Thomas S., Heinrich P. (2006). Biomimetics and Technical Textiles: Solving Engineering Problems with the Help of Nature’s Wisdom. Am. J. Bot..

[15] Matsuo T. (2008). Fibre materials for advanced technical textiles. Text. Prog..

[16] Park S., Jayaraman S. (2003). Smart Textiles: Wearable Electronic Systems. Mrs Bull..

[17] Revaiah R.G., Kotresh T.M., Kandasubramanian B. (2020). Technical textiles for military applications. J. Text. Inst..

[18] Schwarz A., Langenhove L.V., Guermonprez P., Deguillemont D. (2010). A roadmap on smart textiles. Text. Prog..

[19] Willert A., Meuser C., Baumann R.R. (2018). Printed batteries and conductive patterns in technical textiles. Jpn. J. Appl. Phys..

[20] Wu L., Ge Y., Zhang L., Yu D., Wu M., Ni H. (2018). Enhanced electrical conductivity and competent mechanical properties of polyaniline/polyacrylate (PANI/PA) composites for antistatic finishing prepared at the aid of polymeric stabilizer. Prog. Org. Coat..

[21] Machotova J., Kalendova A., Zlamana B., Snuparek J., Palarcik J., Svoboda R. (2020). Waterborne Coating Binders Based on Self-Crosslinking Acrylic Latex with Embedded Inorganic Nanoparticles: A Comparison of Nanostructured ZnO and MgO as Crosslink Density Enhancing Agents. Coatings.

[22] Kaur J., Krishnan R., Ramalingam B., Jana S. (2020). Hydroxyethyl sulfone based reactive coalescing agents for low-VOC waterborne coatings. RSC Adv..

[23] Liu Q., Liao B., Pang H., Lu M.G., Meng Y.Y. (2020). Preparation and characterization of a self -matting coating based on waterborne polyurethane-polyacrylate hybrid dispersions. Prog. Org. Coat..

[24] Wang Y.X., Li C., Zhang X.P., Lin Q.Q., Jiang Y., Yuan J.F., Pan M.W. (2020). Poly(vinylidene chloride)/Poly(chlorotrifluoroethylene-co-acrylates) Composite Latex Coating Cured at Room Temperature Showing an Excellent Corrosion Resistance. Chemistryselect.

[25] Lan T., An R., Liu Z., Li K., Xiang J., Liu G. (2018). Facile fabrication of a biomass-based film with interwoven fibrous network structure as heterogeneous catalysis platform. J. Colloid Interface Sci..

[26] Costentin C., Savéant J.-M. (2019). Molecular approach to catalysis of electrochemical reaction in porous films. Curr. Opin. Electrochem..

[27] Wei Y., Li J., Li Y., Zhao B., Zhang L., Yang X., Chang J. (2017). Research on permeability coefficient of a polyethylene controlled-release film coating for urea and relevant nutrient release pathways. Polym. Test..

[28] Ghani M. (2020). Nanocrystalline cellulose as a biotemplate for preparation of porous titania thin film as a sorbent for thin film microextraction of ketorolac, meloxicam, diclofenac and mefenamic acid. J. Chromatogr. B.

[29] Wei D., Zhou R., Cheng S., Feng W., Li B., Wang Y., Jia D., Zhou Y., Guo H. (2013). Microarc oxidized TiO2 based ceramic coatings combined with cefazolin sodium/chitosan composited drug film on porous titanium for biomedical applications. Mater. Sci. Eng. C.

[30] Xiong X., Xie F., Meng J. (2018). Preparation of superhydrophobic porous coating film with the matrix covered with polydimethylsiloxane for oil/water separation. Prog. Org. Coat..

[31] Chen Y., Krings S., Booth J.R., Bon S.A.F., Hingley-Wilson S., Keddie J.L. (2020). Introducing Porosity in Colloidal Biocoatings to Increase Bacterial Viability. Biomacromolecules.

[32] Ma Y., Davis H.T., Scriven L.E. (2005). Microstructure development in drying latex coatings. Prog. Org. Coat..

[33] Ludwig I., Schabel W., Kind M., Castaing J.-C., Ferlin P. (2007). Drying and film formation of industrial waterborne latices. Aiche J..

[34] Appel L.E., Zentner G.M. (1991). Use of Modified Ethylcellulose Lattices for Microporous Coating of Osmotic Tablets. Pharm. Res..

[35] Hodges I.C., Hearn J. (2001). Reactive Latex Films. Langmuir.

[36] Yang S., Aoki Y., Skeldon P., Thompson G.E., Habazaki H. (2011). Growth of porous anodic alumina films in hot phosphate–glycerol electrolyte. J. Solid State Electrochem..

[37] Ariyanti S., Man Z., Bustam M.A. (2013). Improvement of Hydrophobicity of Urea Modified Tapioca Starch Film with Lignin for Slow Release Fertilizer. Adv. Mater. Res..

[38] Steward P.A., Hearn J., Wilkinson M.C. (1995). Studies on permeation through polymer latex films, I. Films containing no or only low levels of additives. Polym. Int..

[39] Fogden A. (2009). Porous polymer films cast from latex–glucose dispersions. Colloids Surf. A Physicochem. Eng. Asp..

[40] Malik T., Razzaq H., Razzaque S., Nawaz H., Siddiqa A., Siddiq M., Qaisar S. (2019). Design and synthesis of polymeric membranes using water-soluble pore formers: An overview. Polym. Bull..

[41] Sabir A., Shafiq M., Islam A., Khan S., Jamil T., Zahid M., Shafeeq A., Shehzad M.A., Bhatti A., Habib Y. (2015). Influence of polyethylene glycol 600 on cellulose acetate membranes for reverse osmosis desalination. Polym. Res. J..

[42] Calderon-Moreno J.M., Preda S., Predoana L., Zaharescu M., Anastasescu M., Nicolescu M., Stoica M., Stroescu H., Gartner M., Buiu O. (2014). Effect of polyethylene glycol on porous transparent TiO2 films prepared by sol–gel method. Ceram. Int..

[43] Joshi M., Adak B., Butola B.S. (2018). Polyurethane nanocomposite based gas barrier films, membranes and coatings: A review on synthesis, characterization and potential applications. Prog. Mater. Sci..

[44] Zhang C., Lyu P., Xia L., Wang Y., Li C., Xiang X., Dai F., Xu W., Liu X., Deng B. (2018). Regulation of pore morphologies of PU films and thereof water vapor permeability by varying tetrahydrofuran concentration in binary solvent. Polym. Test..

[45] Wang P., Ma J., Wang Z., Shi F., Liu Q. (2012). Enhanced Separation Performance of PVDF/PVP-g-MMT Nanocomposite Ultrafiltration Membrane Based on the NVP-Grafted Polymerization Modification of Montmorillonite (MMT). Langmuir.

[46] Shen Y., Ye J., Yang L., Wu M., Ni T. (2010). Effect of Polyvinylpyrrolidone on the Morphologies of Latex Films and Air Permeability of the Latex Finished Fabrics. J. Donghua Univ. (Engl. Ed.).

[47] Shen Y., Yang L., Ye J., Chen L. (2010). Preparation of Latex Film with Macropores and Their Applications in Pigment Dyeing. J. Donghua Univ. (Engl. Ed.).

[48] Winnik M.A., Feng J. (1996). Latex blends: An approach to zero VOC coatings. J. Coat. Technol..

[49] Lovell P.A., El-Aasser M.S. (1997). Emulsion Polymerization and Emulsion Polymers.

[50] Winnik M.A. (1997). Latex film formation. Curr. Opin. Colloid Interface Sci..

[51] Vandezande G.A., Rudin A. (1996). Film formation of vinyl acrylic latexes; effects of surfactant type, water and latex particle size. J. Coat. Technol..

[52] Vanderhoff J.W., Tarkowski H.L., Jenkins M.C., Bradford E.B. (1967). Theoretical consideration of the interfacial forces involved in the coalescence of latex particles. J. Macromol. Chem..

[53] Vanderhoff J.W., Bradford E.B., Carrington W.K. (1973). The transport of water through latex films. J. Polym. Sci..

[54] Sperry P.R., Snyder B.S., O’Dowd M.L., Lesko P.M. (1994). Role of water in particle deformation and compaction in latex film formation. Langmuir.

[55] Fernando I., Qian T., Zhou Y. (2019). Long term impact of surfactants & polymers on the colloidal stability, aggregation and dissolution of silver nanoparticles. Environ. Res..

[56] Abu-Noqta O.A., Aziz A.A., Usman A.I. (2019). Colloidal Stability of Iron Oxide Nanoparticles Coated with Different Capping Agents. Mater. Today Proc..

[57] Kellaway I.W., Najib N.M. (1980). The adsorption of hydrophilic polymers at the polystyrene-water interface. Int. J. Pharm..

